# Comparative Analysis of Vaginal Microbiota in Women with Breast Cancer in Kazakhstan

**DOI:** 10.31557/APJCP.2021.22.4.1313

**Published:** 2021-04

**Authors:** Farida K. Balmaganbetova, Ainur Amanzholkyzy, Roza E. Nurgaliyeva, Aiman T. Kaldybayeva, Azhar N. Zhexenova

**Affiliations:** 1 *Department of Normal Physiology, West Kazakhstan Marat Ospanov Medical University, Aktobe, Republic of Kazakhstan.*; 2 *Department of Phatophysiology, West Kazakhstan Marat Ospanov Medical University, Aktobe, Republic of Kazakhstan. *

**Keywords:** Oncology, malignant tumour, composition of vaginal microflora, normocenosis, bacterial vaginosis

## Abstract

**Object:**

The relevance of the article is that the breast cancer is a leading oncological disease in women in developed countries and has the highest mortality caused by malignant neoplasms in women. The purpose of the study is to evaluate vaginal microbiota in women with various breast cancer subtypes and compared groups.

**Methods:**

The study involved 278 women with breast cancer, of whom 174 were patients receiving combination therapy; the control group consisted of 104 patients who had had breast cancer 2-4 years ago.

**Results:**

It was found that despite a significant decrease in the total number of *Lactobacillus *spp., there were no statistically significant changes in the numbers of microorganisms in patients with different subtypes of breast cancer. According to the results of the comparative analysis, the representatives of obligate anaerobic *flora Peptostreptococcus* spp. prevailed in vaginal microbiota in luminal A and luminal B subtypes, and the representative of the facultative anaerobic organisms Staphylococcus spp. – in unfavourable outcomes in Her2/Neu+ and triple-negative subtypes.

**Conclusion:**

The observed features of the vaginal microbiota in women with different subtypes of breast cancer require further studies for preventive purposes.

## Introduction

A steady increase in the rates of breast cancer in the world and the Republic of Kazakhstan requires the conduct and continuation of various studies, though many studies have been already dedicated to the problem of breast cancer. Breast cancer is a leading oncological disease in women in developed countries, leaving behind other neoplasms. More than 2,000,000 women (10-18% of all malignant neoplasms) are diagnosed with breast cancer every year worldwide. In the Republic of Kazakhstan, up to 4,000 new cases of this oncological disease are registered annually, while the age of women is constantly decreasing, which affects the most productive, socially active, and reproductive age group of the female population of the city. Therefore, the urgency of the problem is due to the increase in the rates of breast cancer worldwide, in the Commonwealth of Independent States (CIS) countries and the Republic of Kazakhstan. The urgency of this problem is also since breast cancer is a leading cause of mortality in women among malignant neoplasms (Abitova et al., 2018; Morrison et al., 2018).

Breast cancer is a heterogeneous group of tumours that differ in morphology, clinical course, and response to treatment. The study of the correlation between gene expression and immunohistochemical markers in the tumour allowed the identification of several molecular subtypes of breast cancer. Based on an immunohistochemical study of the expression of oestrogen and progesterone receptors (ER and PR), as well as type 2 epidermal growth factor receptor (HER2/Neu, ERbB2), by breast carcinoma cells, breast cancer can be classified into 4 molecular subtypes that differ in the clinical prognosis and response to pharmacotherapy (Jarman et al., 2020; Fernández et al., 2018; Wessels et al., 2018).

Luminal, HER2/Neu+, and triple-negative molecular subtypes of breast cancer are identified. Tumours expressing ER and PR receptors are luminal, and depending on the expression of HER2/Neu, they are divided into luminal A (do not express HER2/Neu) and luminal В (express HER2/Neu). HER2/Neu+ tumours are those with overexpression of Her2/Neu and no ER and PR. Tumours negative for 3 of the aforementioned characteristics belong to triple-negative breast cancer. It is known that luminal types are associated with a less aggressive course and a favourable prognosis compared to HER2/Neu+ and triple-negative breast cancer subtypes (Chen et al., 2019). A triple-negative subtype is associated with a high frequency of breast cancer (BRCA1) mutations, aggressive course, lack of response to chemotherapy and hormone therapy, and low survival rates (Alizadehmohajer et al., 2020; Babyshkina et al., 2017).

To determine biological subtypes of breast cancer, an immunohistochemical study is carried out where oestrogen and progesterone receptors, HER2 overexpression, or C-ERB B2 oncogen and Ki-67 cell proliferation marker amplifications are indicators of tumour malignancy. Based on the results, luminal A, luminal B, HER2-positive and triple-negative breast cancer subtypes are identified. The normal microflora of the vagina is a combination of normoflora and oportunistic pathogenic microflora, which provide a certain pH medium for optimal vital activity of microorganisms and creates conditions for adequate functioning of nonspecific immunity in the vagina (Shatova et al., 2017; Hammerl et al., 2018). Microbiota is a kind of a sensitive indicator that responds with quantitative and qualitative changes to any changes in the external and internal environment. A change in the number of a particular type of microorganisms in the biotope or the appearance of bacteria not characteristic of the given habitat is a signal of adaptive or irreversible changes in the corresponding element of the microecological system. The functioning and coordinated interactions between all the elements of the microbial system of the host are ensured by the coordinated activity of the immune and endocrine systems, reflect their functional state, and depend on the factors of both the internal and external environment. Failure of one of these elements invariably destroys microecology in the vagina, which can result in inflammatory processes in the genitals, both local and systemic, which significantly affects the development and progression of the tumour (Akram et al., 2017; Balmaganbetova et al., 2019).

The Human Microbiome Project, initiated by the US National Institute of Health, investigated 900 complete microbial genomes in 300 healthy volunteers. According to the Project estimates, microorganisms give the human body another 8 million genes (Shamsi and Pirayesh Islamian, 2017). Apart from bacterial flora, human papillomavirus (HPV) in the vagina is one of the determinants in the development of breast cancer, which indicates that the genotypes of HPV 44; 45; 53; 73 are often found and associated with different grades of dysplasia of high oncogenic risk (Ljungma et al., 2018). Chemotherapy and endocrine therapy affect healthy vaginal microbiota in women with breast cancer, which undoubtedly affects their quality of life (Baxevanis et al., 2019).

## Materials and Methods

The study was carried out in the city of Aktobe of the Republic of Kazakhstan, in the Medical Centre of the Marat Ospanov West Kazakhstan Medical University, as part of a grant by the Ministry of Science and Education of the Republic of Kazakhstan for the scientific and technical project No. 0118РК01065, during 2018-2020. The study involved 278 women with breast cancer divided into 4 subtypes (1, 2, 3, 4) according to molecular classification using immunohistochemical analysis (IGH): 1 – luminal A – 147 (53%); 2 – luminal B – 57 (21%); 3 – Her2/Neu+ – 26 (9%); 4 – triple-negative – 48 patients (17%). According to the molecular classification, the prognosis of the disease is more favourable for luminal A and luminal B subtypes, compared to other subtypes.

The women enrolled in the study were further divided into two observation groups: the study group included 174 patients (62.5%) who received combination therapy (chemotherapy and hormone therapy); the control group consisted of 104 patients (37.4%) who had breast cancer 2-4 years ago, registered with a mammologist-oncologist. Patients signed informed consent to participate in the study.

The qualitative and quantitative composition of the vaginal flora in women with breast cancer was determined using the Femoflor reagent kit. The scrapings from the posterolateral wall of the vagina using a urogenital probe were placed in special transport media for bioassays containing isotonic saline solution with preservatives for scrapings, which were subsequently delivered to the polymerase chain reaction (PCR) laboratory of the Scientific and Practical Centre (SPC) of the Marat Ospanov West Kazakhstan Medical University, where the method of PCR in real-time was used for qualitative and quantitative assessment of vaginal microflora. The content of microorganisms was expressed as a decimal logarithm of the absolute amount of deoxyribonucleic acid (DNA). A relative number of bacteria was calculated as a logarithm of the ratio of the determined microorganism to the total bacterial mass. The state of normocenosis was determined according to the content of Lactobacillus spp.; the content of Lactobacillus spp. of 106-108 was regarded as a state of normocenosis. Values of 104-105 corresponded to the state of moderate dysbiosis and less than 104 – to the state of severe dysbiosis. The content of facultative anaerobes over 104 was regarded as anaerobic dysbiosis and nonspecific vaginitis. An increase in obligate anaerobes of more than 104 indicated bacterial vaginosis. Candida spp. more than 103 indicated vaginal candidiasis, as well as an increase in mycoplasma representatives: Mycoplasma hominins, Mycoplasma genitalium, and Ureaplasma more than 103 indicated mycoplasmosis and nonspecific vaginitis.

Statistical processing of this analysis was carried out using the licensed program Statistica 10.0. Methods of descriptive statistics with the calculation of central trends and their magnitude were used for quantitative variables and the percentage of the parameter for qualitative data. The results were expressed as the median and interquartile range, arithmetic means, and standard deviation. To compare the studied groups by quantitative variables, the non-parametric Mann-Whitney test was used for independent samples and the Kruskal-Wallis test for multiple comparisons of variables. Differences were considered statistically significant at p<0.05. A detailed in-depth analysis of the ratio of intergroup and intragroup variances was carried out posterior using the Fisher F-test according to the least significant difference (LSD). The comparison was made with the “right” quantile of distribution. The ratio of intergroup and intragroup variances has an F-distribution (Fisher distribution) and is determined using the Fisher’s F-test: one-factor and multi-factor analysis of variance (one or more independent variables).

## Results

The results of the vaginal microbiota, which are shown in Table 1, in Luminal A subtype: representative of normoflora *Lactobacillus* spp. – 10^5.7^ [10^4.4^-10^6.5^] – below normal, which indicates dysbiosis. The representatives of facultative anaerobic flora: *Enterobacterium* spp. 10^3.9^ [10^3.3^-10^4.9^], *Streptococcus* spp. 10^3.6^ [10^2.8^-10^4.5^], *Staphylococcus* spp. 10^3.3^ [10^3.1^-10^3.9^] – correspond to normal. Representatives of obligate anaerobic flora: *Sneathia *spp. 10^3.9^ [10^3.6^ -10^5.1^], *Mobyluncus* spp. 10^3.8^ [10^3.3^-10^4.4^], Megasphaera spp. 10^3.5^ [10^3.1^-10^4.7^], *Atopobiumvaginae* 10^3.3^ [10^2.2^-10^4.9^] – correspond to normal, and other representatives of the same flora: *Gardnerellavaginalis* 10^4.6 ^[10^3.5^-10^5.6^], Eubacterium spp. 10^4.7^ [103.6-10^5.6^], Lachnobacterium spp. 10^4.1^ [10^3.2^-10^5.1^], *Peptostreptococcus* spp. 10^4.5^ [10^3.6^-10^5.7^] – increased, which indicates the presence of bacterial vaginosis (anaerobic dysbiosis). Representative of yeasts:* Candida *spp. 10^3.4^ [10^2.4^-10^4.7^] – higher than normal, indicating vaginal candidiasis. Representatives of *mycoplasmas: Mycoplasma* hominis 10^3.3^ [10^2.4^-10^4.7^], *Mycoplasma genitalium* 10^3.4^ [10^2.1^-10^4.2^], *Ureaplasma* (*urealyticum + parvum*) 10^3.6^ [10^2.6^-10^4,7^] – increased, which indicate the presence of *mycoplasmosis, ureaplasmosis, nonspecific vaginitis*, and *urethritis*.

In luminal B subtype: *Lactobacillus* spp. 10^5.0^ [10^3.8^-10^6.7^] – below normal. *Enterobacterium* spp. 10^4.9^ [10^3.6^-10^6.1^] – increased, as well as Gardnerella vaginalis 10^4.1^ [10^3.4^-10^5.1^] and *Eubacterium *spp. 10^4.3^ [10^3.4^-10^5.6^] – slightly increased. In this group, Mycoplasma hominis was 10^3.4^ [10^2.5^-10^4.4^], *Mycoplasma genitalium* 10^3.5^ [10^2.1^-10^4.7^],* Ureaplasma* 10^3.7^ [10^2.5^-10^4.3^] – increased.

In the subtype Her2/Neu+, the microbiota was as follows:* Lactobacillus* spp. – 10^4.2 ^[10^3.5^-10^6.4^] – significantly below normal; *Megasphaera* spp. 10^4.3^ [10^3.1^-10^5.1^], *Lachnobacterium* spp. 10^4.3^ [10^3.3^-10^5.2^], *Mobyluncus* spp. 10^4.2^ [10^3.9^-10^4.7^], *Gardnerella vaginalis* 10^4.1^ [10^3.5^-10^5.2^], *Eubacterium spp*. 10^4.1 ^[10^2.8^-10^5.3^] – slightly increased, and *Peptostreptococcus *spp. 10^5.6^ [10^5.3^-10^6.4^] – significantly increased. *Candida *spp. 10^3.3^ [10^3.1^-10^3.6^], *Mycoplasma hominis* 10^3.1^ [10^1.8^-10^3.5^], *Ureaplasma* 10^3.6^ [10^3.2^-10^4.4^] – slightly increased.

In triple-negative subtype normoflora was reduced: *Lactobacillus* spp. 10^5.3^ [10^4.7^-10^6.9^]. *Gardnerella vaginalis* 10^5.2^ [10^3.5^-10^6.4^] – significantly increased. *Eubacterium* spp. 10^4.1^ [10^3.2^-10^5.4^], *Lachnobacterium* spp. 10^4.4^ [10^3.5^-10^5.6^] – slightly increased; *Candida* spp. 10^3.4^ [10^2.4^-10^3.9^], *Mycoplasma hominis* 10^3.8^ [10^3.2^-10^6.1^], *Mycoplasma genitalium* 10^3.3^ [10^2.1^-10^5.4^], *Ureaplasma* 10*3.8* [10^3.3^-10^4.8^] – slightly increased (Table 1).

For the representative of the same group of *obligate anaerobes*, *Lachnobacterium* spp. in luminal B and triple-negative subtypes (p = 0.01), in the marginal areas between favourable and unfavourable outcomes, a significant increase in the logarithm of the ratio of the microorganism* Lachnobacterium* spp. was observed, which was sensitive to a decrease in non-specific immunity (Figure 2).

The following comparative analysis shows that the representative of the facultative anaerobes,* Staphylococcus *spp. showed a significant difference in adverse outcomes in Her2/Neu+ and triple-negative subtypes (p = 0.02); which is indicated by an increase in the mean value of the logarithm of the ratio of the determined microorganism in triple-negative subtype (Figure 3).

Among all the representatives of *Mycoplasma*, *Mycoplasma genitalium* demonstrates the same results as presented above for Staphylococcus spp. in the subtypes Her2/Neu+ and triple-negative (p = 0.04) (Figure 4).

**Table 1:** Parameters of Vaginal Microbiota in Various Subtypes of Breast Cancer

Parameter/microorganism	Molecular subtypes of breast cancer according to IGH			
	Luminal A	Luminal В	Her2/Neu+	Triple-negative
Normoflora representative				
Lactobacillus spp.	10^5.7^ [10^4.4^-10^6.5^]	10^5.0^ [10^3.8^-10^6.7^]	10^4.2 ^[10^3.5^-10^6.4^]	10^5.3^ [10^4.7^-10^6.9^]
Facultative anaerobes				
Enterobacterium spp.	10^3.9^ [10^3.3^-10^4.9^]	10^4.9^ [10^3.6^-10^6.1^]	10^5.2^ [10^5.1^-10^5.2^]	10^3.1^ [10^2.9^-10^4.4^]
Streptococcus spp.	10^3.6^ [10^2.8^-10^4.5^]	10^3.6^ [10^3.5^-10^3.8^]	10^3.9^ [10^3.8^-10^3.9^]	10^3.3^ [10^2.6^-10^5.6^]
Staphylococcus spp.	10^3.3^ [10^3.1^-10 ^3.9^]	10^3.4^ [10^3.1^-10^3.9^]	10^2.8^ [10^2.1^-10 ^3.5^]	10^3.8^ [10^3.8^-10^4.5^]
Obligate anaerobes				
Sneathia spp.	10^3.9^ [10^3.6^-10^5.1^]	10^3.7^ [10^3.2^-10^4.1^]	10^3.8^ [10^3.7^-10^3.9^]	10^3.5^ [10^2.8^-10^5.7^]
Mobyluncus spp.	10^3.8^ [10^3.3^-10^4.4^]	10^3.8^ [10^3.5^-10^4.5^]	10^4.2^ [10^3.9^-10^4.7^]	10^3.8^ [10^3.2^-10^5.2^]
Megasphaera spp.	10^3.5 ^[10^3.1^-10^4.7^]	10^3.9^ [10^3.4^-10^5.3^]	10^4.3^ [10^3.1^-10^5.1^]	10^3.8^ [10^3.4^-10^4.2^]
Atopobium vaginae	10^3.3^ [10^2.2^-10^4.9^]	10^3.7^ [10^2.8^-10^4.9^]	10^3.9^ [10^1.0^-10^6.3^]	10^3.8^ [10^3.1^-10^5.0^]
Gardnerella vaginalis	10^4.6^ [10^3.5^-10^5.6^]	10^4.1^ [10^3.4^-10^5.1^]	10^4.1^ [10^3.5^-10^5.2^]	10^5.2^ [10^3.5^-10^6.4^]
Eubacterium spp.	10^4.7^ [10^3.6^-10^5.6^]	10^4.3^ [10^3.4^-10^5.6^]	10^4.1^ [10^2.8^-10^5.3^]	10^4.1^ [10^3.2^-10^5.4^]
Lachnobacterium spp.	10^4.1^ [10^3.^2-10^5.1^]	10^3.3^ [10^2.9^-10^4.3^]	10^4.3^ [10^3.3^-10^5.2^]	10^4.4^ [10^3.5^-10^5.6^]
Peptostreptococcus spp.	10^4.5^ [10^3.6^-10^5.7^]	10^3.4^ [10^3.0^-10^4.1^]	10^5.6^ [10^5.3^-10^6.4^]	10^4.0^ [10^3.1^-10^5.3^]
Yeasts				
Candida spp.	10^3.4^ [10^2.4^-10^4.7^]	10^2.9^ [10^2.1^-10^4.4^]	10^3.3^ [10^3.1^-10^3.6^]	10^3.4^ [10^2.4^-10^3.9^]
Mycoplasma				
Mycoplasma hominis	10^3.3^ [10^2.4^-10^4.7^]	10^3.4^ [10^2.5^-10^4.4^]	10^3.1^ [10^1.8^-10^3.5^]	10^3.8^ [10^3.2^-10^6.1^]
Mycoplasma genitalium	10^3.4^ [10^2.1^-10^4.2^]	10^3.5^ [10^2.1^-10^4.7^]	10^2.1^ [10^2.1^-10^2.3^]	10^3.3^ [10^2.1^-10^5.4^]
Ureaplasma (urealyticum + parvum)	10^3.6^ [10^2.6^-10^4.7^]	10^3.7^ [10^2.5^-10^4.3^]	10^3.6^ [10^3.2^-10^4.4^]	10^3.8^ [10^3.3^-10^4.8^]

Me, median, 25% is the lower quartile, 75% is the upper quartile

**Figure 1:**
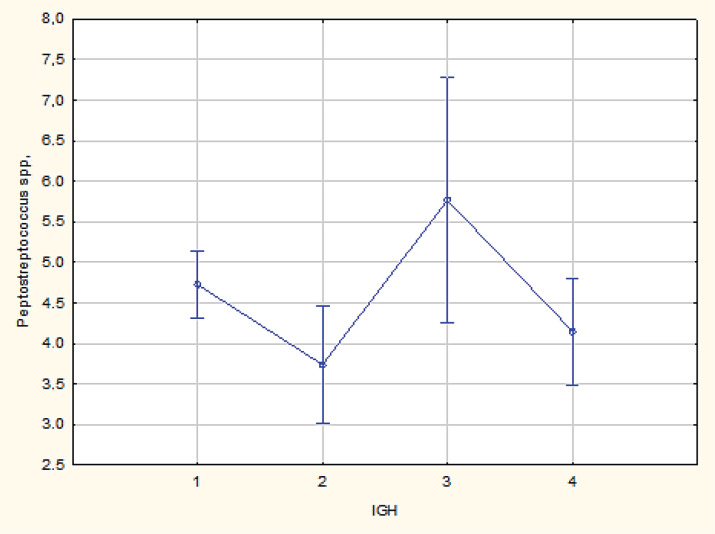
Comparative Analysis of Peptostreptococcus spp. in Breast Cancer Subtypes

**Figure 2:**
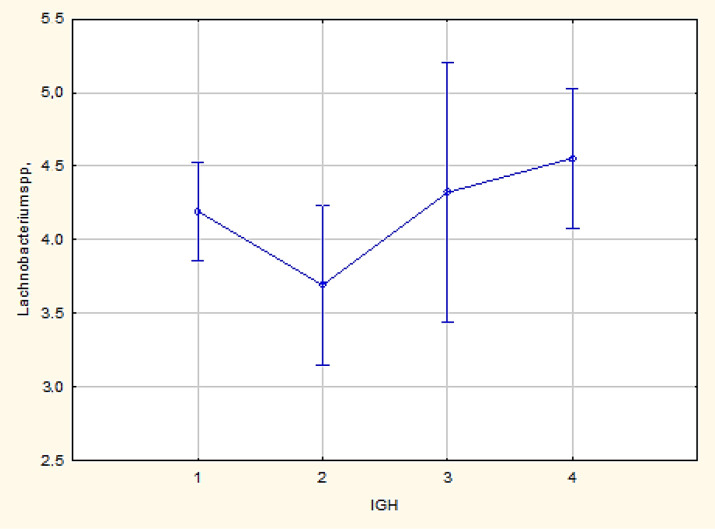
Comparative Analysis for Lachnobacterium spp. in Luminal B and Triple-Negative Subtypes

**Figure 3:**
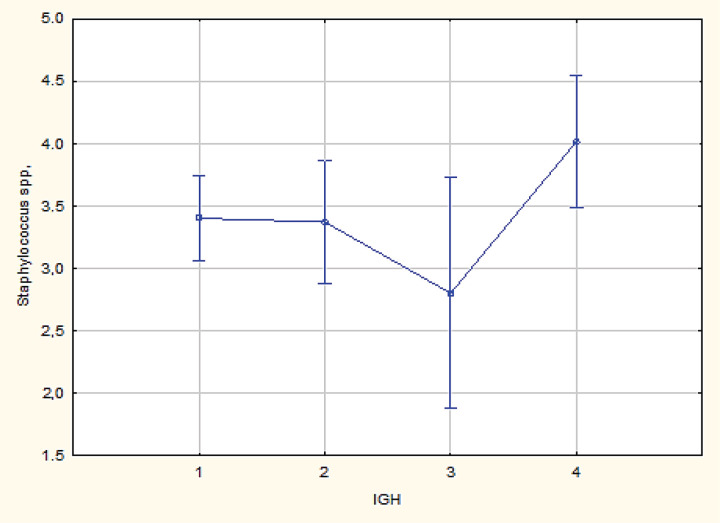
Comparative Analysis for Staphylococcus spp. in Her2/Neu+ and Triple-Nnegative Subtypes

**Figure 4:**
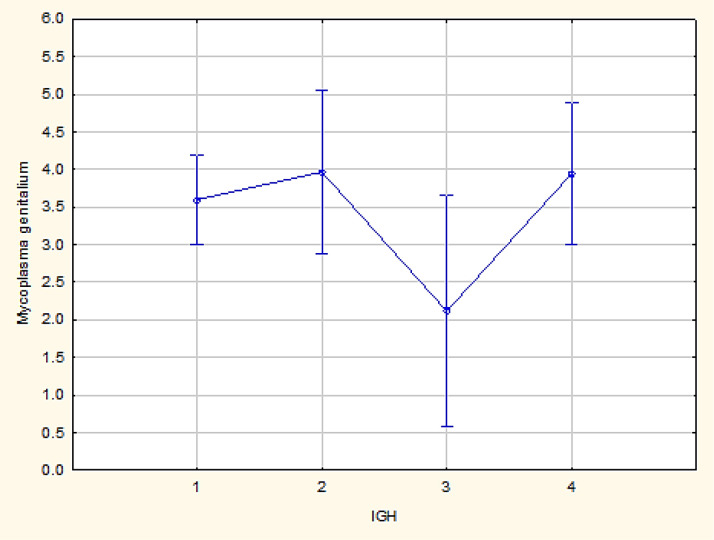
Comparative analysis of Mycoplasma Genitalium in Her2/Neu+ and Triple-Negative Subtypes

## Discussion

Chemotherapy and estrogen therapy in women with breast cancer inhibit ovarian function and decrease the level of sex hormones: estrogen and progesterone, which subsequently result in inflammatory processes of the vagina – bacterial vaginosis (BV), aerobic vaginitis (AB), and vulvovaginal candidiasis (VVC). All these diseases are associated with genitourinary syndrome of menopause, which is a common condition in patients with breast cancer. It is also known that urogenital syndrome of menopause is associated with a decrease in estrogen secretion, which leads to depletion of lactobacilli and an increase in vaginal pH, followed by colonization with enterobacteria (Hashmi et al., 2018).

A randomized, placebo-controlled, double-blind experimental study conducted at the Department of Obstetrics and Gynaecology, Vienna Medical University (Austria) showed that *Lactobacillus* spp. were absent in vaginal microbiota in women with breast cancer receiving chemotherapy, which was regarded as not just a simple deficit, but as a pathological condition. Due to chemotherapy and oestrogen therapy, patients with BV have an increased risk of lack or reduction in vaginal lactobacilli (Ho and Yu, 2019). The same results were obtained in this study.

According to Kim et al., (2020), most patients with breast cancer had no *Lactobacillus* spp., only 14% were present, which indicated that the majority of patients with an imbalance of vaginal flora in this group had aerobic vaginitis, and not bacterial vaginosis. In this study, patients with breast cancer and *Lactobacillus* spp. deficiency were 55%, therefore 45% of patients had two types of the above-mentioned inflammatory diseases of the vagina.

The findings of Podgornaya et al., (2017) show that bacterial vaginosis has a recurrent nature and even in periods of remission, there is a potential risk of exacerbation, which is manifested as a state of moderate dysbiosis, predominance of *Lactobacillus* iners, and certain types of anaerobes. According to Komohara et al., (2016), the composition of vaginal microbiota in patients with vulvovaginitis after radical treatment and adjuvant therapy for breast cancer was characterized by a predominance of aerobic representatives of *Enterobacterium* spp., *Staphylococcaceae*, which is also confirmed by the results, i.e., an increase in *Staphylococcus* spp. in the triple-negative subtype. In breast cancer, the observed microbiota was more pathogenic.

In conclusion, thus, the results of the study showed that breast cancer luminal A subtype is the most common among all examined patients and has the most favourable prognosis compared to other subtypes. As an imbalance in the normal vaginal microbiota can cause infectious and inflammatory diseases of the female reproductive system, as well as during adjuvant therapy for breast cancer, it is difficult to overestimate the value of *Lactobacillus* spp. in female reproductive health, but the role of *Lactobacillus* spp. in maintaining the normocenosis of the vagina remains unclear. The above data shows that the representatives of obligate anaerobic flora* Peptostreptococcus* spp. and *Lachnobacterium* spp. tend to have significant multiple intergroup comparisons in the proposed subtypes.

The system of vaginal microbiota in women with different subtypes of breast cancer requires further studies to determine the relationships between microbiome and tumour genesis, which will result in an understanding the mechanism of the influence of microbiota on tumour growth and changes in the microbiome under the influence of tumour growth. Therefore, the identified features of vaginal microbiota in women with different subtypes of breast cancer should be investigated in detail for preventive purposes.

## Author Contribution Statement

Farida Balmaganbetova, Ainur Amanzholkyzy and Roza Nurgaliyeva processed the experimental data, performed the analysis, drafted the manuscript and designed the figures. Aiman Kaldybayeva and Azhar Zhexenova aided in interpreting the results and worked on the manuscript. All authors discussed the results and commented on the manuscript.
